# Blood Flow Velocity Pulsatility and Arterial Diameter Pulsatility Measurements of the Intracranial Arteries Using 4D PC-MRI

**DOI:** 10.1007/s12021-021-09526-7

**Published:** 2021-05-21

**Authors:** Kees M. van Hespen, Hugo J. Kuijf, Jeroen Hendrikse, Peter R. Luijten, Jaco J. M. Zwanenburg

**Affiliations:** 1 Center for Image Sciences, UMC Utrecht, Utrecht, The Netherlands; 2 Image Sciences Institute, UMC Utrecht, Utrecht, The Netherlands; 3 Department of Radiology, UMC Utrecht, Utrecht, The Netherlands

**Keywords:** 4D PC-MRI, Blood flow velocity pulsatility, Arterial distensibility, Intracranial arteries, Arterial stiffness, Diameter pulsatility

## Abstract

4D phase contrast magnetic resonance imaging (PC-MRI) allows for the visualization and quantification of the cerebral blood flow. A drawback of software that is used to quantify the cerebral blood flow is that it oftentimes assumes a static arterial luminal area over the cardiac cycle. Quantifying the lumen area pulsatility index (aPI), i.e. the change in lumen area due to an increase in distending pressure over the cardiac cycle, can provide insight in the stiffness of the arteries. Arterial stiffness has received increased attention as a predictor in the development of cerebrovascular disease. In this study, we introduce software that allows for measurement of the aPI as well as the blood flow velocity pulsatility index (vPI) from 4D PC-MRI. The internal carotid arteries of seven volunteers were imaged using 7 T MRI. The aPI and vPI measurements from 4D PC-MRI were validated against measurements from 2D PC-MRI at two levels of the internal carotid arteries (C3 and C7). The aPI and vPI computed from 4D PC-MRI were comparable to those measured from 2D PC-MRI (aPI: mean difference: 0.03 (limits of agreement: −0.14 – 0.23); vPI: 0.03 (−0.17–0.23)). The measured blood flow rate for the C3 and C7 segments was similar, indicating that our proposed software correctly captures the variation in arterial lumen area and blood flow velocity that exists along the distal end of the carotid artery. Our software may potentially aid in identifying changes in arterial stiffness of the intracranial arteries caused by pathological changes to the vessel wall.

## Introduction

Arterial stiffness has received increased attention as a predictor in the development of cerebrovascular disease (Dalgleish et al., 2006; Zarrinkoob et al., 2016), and is linked to pathological conditions such as hypertension (Boutouyrie et al., 2002), diabetes (Muhammad et al., 2017) and end-stage renal disease (Blacher et al., 1998). Increased arterial stiffness results in a more pulsatile flow to the smaller arteries (Mitchell et al., 2011; O’Rourke & Hashimoto, 2007; Shirwany & Zou, 2010; Webb et al., 2012), where the excessive pulsatility could induce damage to the microcirculation (Schnerr et al., 2017), leading to observable damage such as microbleeds (Zhai et al., 2018), lacunar infarcts (Chuang et al., 2016) and white matter hyperintensities (Aribisala et al., 2014). In addition, an increase in arterial stiffness and blood flow velocity pulsatility have been linked to cognitive impairment (Mitchell et al., 2011; Muhire et al., 2019).

Traditionally, quantitative measurements on the blood flow are for example performed using transcranial Doppler ultrasound (Markus, 1999). Doppler ultrasound, although cheap and widely available, has several drawbacks including operator dependence and limited penetration of the ultrasound signal in the skull (Markus, 1999). More recently, phase contrast (velocity) magnetic resonance imaging (PC-MRI) has been used to quantify the blood flow (Dunås et al., 2018; Schubert et al., 2011). Phase contrast MRI relies on the accrual of phase of the MR signal for moving spins. This accrual is proportional to the velocity of the moving spins, which can therefore be calculated from the phase of the MR signal. 2D PC-MRI, where blood flow velocity is measured over time in a 2D plane, is dependent on operator skill, and is limited to measurements at a single position along an artery. In contrast, 4D PC-MRI sequences, where blood flow velocity is measured over time in a 3D volume, do not suffer from the disadvantages of TCD and 2D PC-MRI, and allow for 3D blood flow quantification of all vessels within a 3D volume. Although 4D PC-MRI is very time-consuming, ongoing developments in MRI, including parallel imaging (Pruessmann et al., 1999) and compressed sensing (Gottwald et al., 2020; Peper et al., 2020) have enabled implementations with practically feasible scan times.

Several image processing tools have been proposed to interpret and analyze 4D PC-MRI data. Most tools are optimized for cardiac blood flow analysis (Köhler et al., 2019; Sinha et al., 2012). Few methods focus on blood flow analysis in the intracranial arteries. Schrauben et al. have proposed a centerline tracking and segmentation method for 4D PC-MRI data of the intracranial arteries (Schrauben et al., 2015). Their segmentation method has later been implemented for patients with intracranial stenosis (Vali et al., 2019), where center line tracking as well as lumen segmentation was performed. One drawback of these image processing tools for blood flow analysis of the intracranial arteries is that they assume a static luminal area across the cardiac cycle. The change in luminal area due to a rise in distending pressure over the cardiac cycle, can however provide insight in the arterial stiffness and provide -in combination with estimations of the pulse pressure- commonly used indices such as arterial distensibility or arterial compliance (O’Rourke & Hashimoto, 2007; Oliver & Webb, 2003).

In this study, we present software for measuring the intracranial blood flow velocity pulsatility as well as the lumen area pulsatility, as measure for the arterial stiffness, from 4D PC-MRI images. Additionally, we validated our blood flow velocity pulsatility and lumen area pulsatility measurements with measurements from 2D PC-MRI.

## Methods

For the quantification of the blood flow velocity pulsatility and lumen area pulsatility, image processing software was written in MeVisLab 3.1.1 (Fraunhofer Mevis, Germany (Ritter et al., 2011)) for analysis of the 4D PC-MRI images. The image processing tool, available from https://github.com/keesvanhespen/DampingGUI (instructions for use in Readme.md file), ran on a standard workstation (Intel Xeon E-1650v3, 32gb RAM). Measurements on the 4D PC-MRI images were validated against measurements performed on 2D PC-MRI images.

### 4D PC-MRI Image Processing

**Fig. 1:**
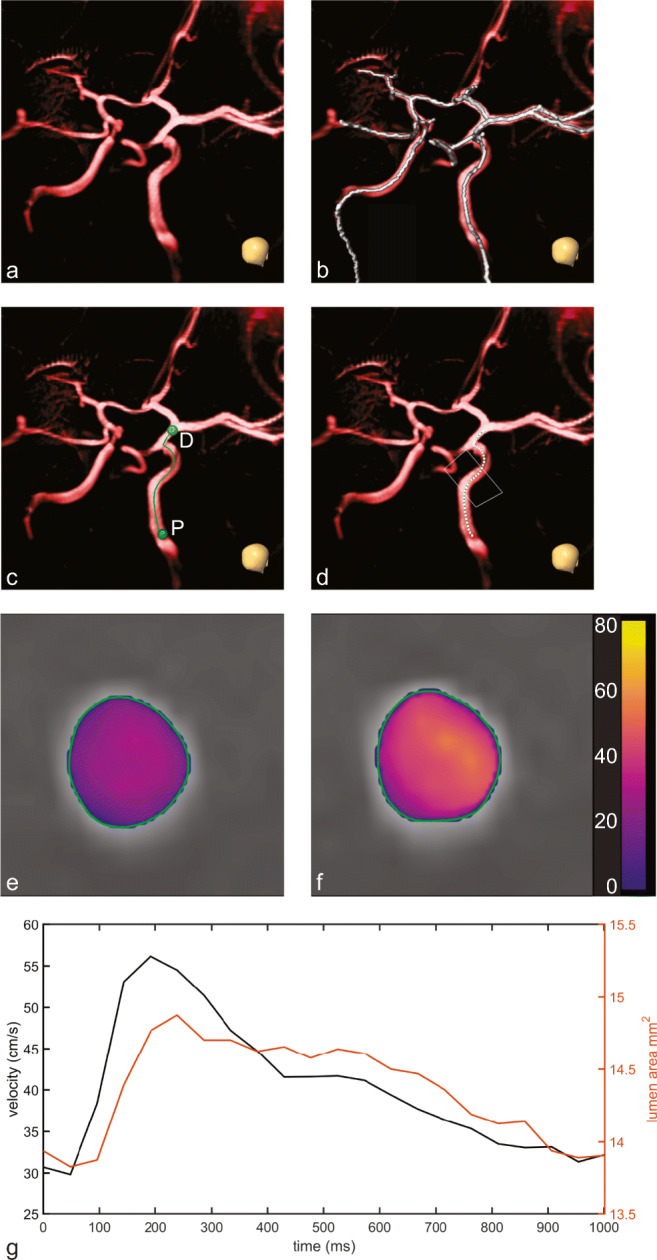
**Blood flow velocity pulsatility and lumen area pulsatility measurement pipeline. a** 3D rendering of T1-weighted image, thresholded to show the circle of Willis arteries. Skull inset in the bottom right corner shows the orientation (coronal view of the circle of Willis in anterior direction). **b** Projected center line skeleton. Note that the skeleton extends in the extracranial carotids and the basilar artery, that are not rendered from the T1-weighted image. **c** Selected start- and end locations as P (proximal), and D (distal). The center line is generated between these points from the skeleton. **d** Measurement locations, given by the white dots along the center line. The white rectangle corresponds to the multi-planar reconstruction (MPR) slice given in **e** and **f**, which is located at the end of the C3 segment. The detected luminal area for this MPR slice and timepoint is given by the green outline, with the velocity in cm/s projected in color for the end-diastolic (**e**) and systolic (**f**) cardiac phases. Mean velocities and lumen areas over the cardiac cycle for this MPR slice are given in **g**

To extract the blood flow velocity pulsatility and lumen area pulsatility measures, several preprocessing steps were performed. Initially, a bias field correction was performed by fitting a first-degree polynomial surface through the time average of stationary voxels in the 4D PC-MRI phase images (Lankhaar et al., 2005). Additionally, phase unwrapping was applied to the 4D PC-MRI phase images. The 4D PC-MRI images were registered to the T1-weighted images, to compensate for subject displacement between scans. A center line of the intracranial arteries was acquired by applying a vesselness filter to the 3D T1w images, and subsequently performing a distance transform skeletonization of the filtered image (Fig. 1b) (Frangi et al., 1998). Manual start- and end positions were chosen on the skeleton, to select the vessel segment on which to evaluate the blood flow velocity pulsatility and lumen area pulsatility (Fig. 1c).

Along the selected segment a multi-planar reconstruction (MPR) was created of the 4D PC-MRI magnitude and phase images in a region of 10 × 10 mm^2^ around the center line (Fig. 1d). The three directional velocity encoded phase images were combined to compute the blood flow velocity component perpendicular through each slice of the MPR.

For each MPR slice and cardiac phase, an isocontour was automatically drawn at the arterial lumen-background boundary at the full-width-at-half-maximum (FWHM) intensity value on the PC-MRI magnitude image, computed per cardiac phase image (Fig. 1e). The FWHM intensity value was calculated from a masked image using Otsu’s method, separating the arterial lumen from the background (Otsu, 1979). The isocontour seed was initialized at the estimated radius of the vessel computed from a rough tubular tracking method applied on the MPR image stack.

The blood flow velocity pulsatility index (vPI) and lumen area pulsatility index (aPI) are flow parameters that are represented by the difference in maximum and minimum velocity/lm area over the cardiac cycle, normalized by the mean velocity/minimum lumen area. A higher index indicates a larger variation of velocity/lm area over the cardiac cycle, whereas an index of 0 indicates that velocity/lm area is stationary over the cardiac cycle. A high vPI has been linked with white matter hyperintensities, cerebral atrophy, and cognitive impairment (Aribisala et al., 2014; Mitchell et al., 2011; Wåhlin et al., 2012). In healthy young volunteers, vPI around 0.84 have been reported for the proximal part of the ICA, with lower values more distally in the vascular tree (van Tuijl et al., 2020; Zarrinkoob et al., 2016). With increasing age and/or underlying cerebrovascular disease, higher vPI can be expected (Zarrinkoob et al., 2016). If the pulse pressure associated with the diameter pulsations is known, the arterial distensibility D can be computed from the aPI as: $D=\frac{aPI}{\partial P}$, with *∂P* the change in pulse pressure over the cardiac cycle (O’Rourke et al., 2002). The arterial distensibility is an established marker for quantifying the arterial stiffness (Segers et al., 2020). The vPI was computed for each MPR slice as:

$$ vPI=\frac{\max \left({v}_{mean}\right)-\min \left({v}_{mean}\right)}{\mathrm{mean}\left({v}_{mean}\right)}, $$

where *v*_*mean*_ is the mean velocity computed within the drawn isocontour for each cardiac phase. The aPI was measured in a similar way as:

$$ aPI=\frac{\max (A)-\min (A)}{\min (A)}, $$

with *A*, the cross-sectional luminal area over the cardiac cycle.

### 2D PC-MRI Image Processing

The processing and analysis of the 2D PC-MRI images was performed relatively similar to that of the 4D PC-MRI images. A manual seed point was generated in the center of the vessel lumen on the 2D PC-MRI magnitude image. Hereafter, isocontours were drawn automatically at the FWHM intensity value between arterial lumen and background for all cardiac phases. Otsu’s thresholding was performed in a region of 10 × 10 mm surrounding the seed point, to separate the arterial lumen from the background, and to subsequently calculate the FWHM intensity value. The vPI was calculated within the isocontours from the 2D PC-MRI phase images, and the aPI was calculated from the cross-sectional luminal area.

### Study Participants

For validation of our software, MR image data was acquired from seven healthy volunteers (age range: 23–28 years, 3 males), which were included in this study after obtaining written informed consent. Data acquisition was approved by the local institutional review board.

### MRI Acquisition

MR examinations were performed on a 7 T MR scanner (Philips, Best, the Netherlands), using an 8-channel transmit coil and a 32-channel receive head coil (Nova Medical, Inc., Wilmington, MA, USA). A 3D T1-weighted TFE image was acquired for anatomical reference. A retrospectively-gated 4D PC-MRI acquisition (acquired voxel size: 0.78 × 0.78 × 0.8 mm^3^, field of view: 250 × 250 × 24.8 mm^3^, reconstructed cardiac phases: 22, flip angle: 15 degrees, repetition time (TR) = 4.51 ms; echo time (TE) = 2.3 ms, SENSE: 3, velocity encoding (v_enc_): 100 cm/s) was angulated to include the intracranial parts of the carotid arteries. The 4D PC-MRI scan was acquired for RL, AP and FH velocity encoding separately. The associated scan duration was three times 5 min and 10 s for a heart rate of 60 bpm. For validation purposes, two retrospectively-gated 2D PC-MRI acquisitions were performed (acquired voxel size: 0.19 × 0.19 × 3 mm^3^, field of view: 250 × 250 × 3 mm^3^, reconstructed cardiac phases: 25, flip angle: 50 degrees, repetition time (TR) = 17 ms; echo time (TE) = 4.2 ms, SENSE: 2, v_enc_: 120 cm/s), where one was angulated at the C3 level (lacerum segment of the internal carotid artery) and one at the C7 level (terminal segment that connects to the circle of Willis) of the internal carotid artery, given the classification by Bouthillier (Bouthillier et al., 1996). The C3 and C7 locations were chosen, as recent work has shown considerable difference in both area and velocity pulsations between these two segments (van Tuijl et al., 2020).The scan duration for the 2D PC-MRI acquisition was 2 min and 6 s for a heart rate of 60 bpm.

### Experimental Setup

We validated the vPI and aPI measured from the 4D PC-MRI images, against measurements performed on 2D PC-MRI images at the same anatomical locations. Additionally, we measured the preservation of blood flow rate between the C3 and C7 segments, given the mean blood velocity and arterial lumen area. Additionally, we compared the blood flow rate measured from 4D PC-MRI with measurements from 2D PC-MRI.

As an example, we also show the vPI and aPI for one volunteer for a large part of the circle of Willis, including the internal carotid arteries, anterior cerebral arteries, middle cerebral arteries, posterior communicating arteries/posterior cerebral arteries.

## Results

**Fig. 2:**
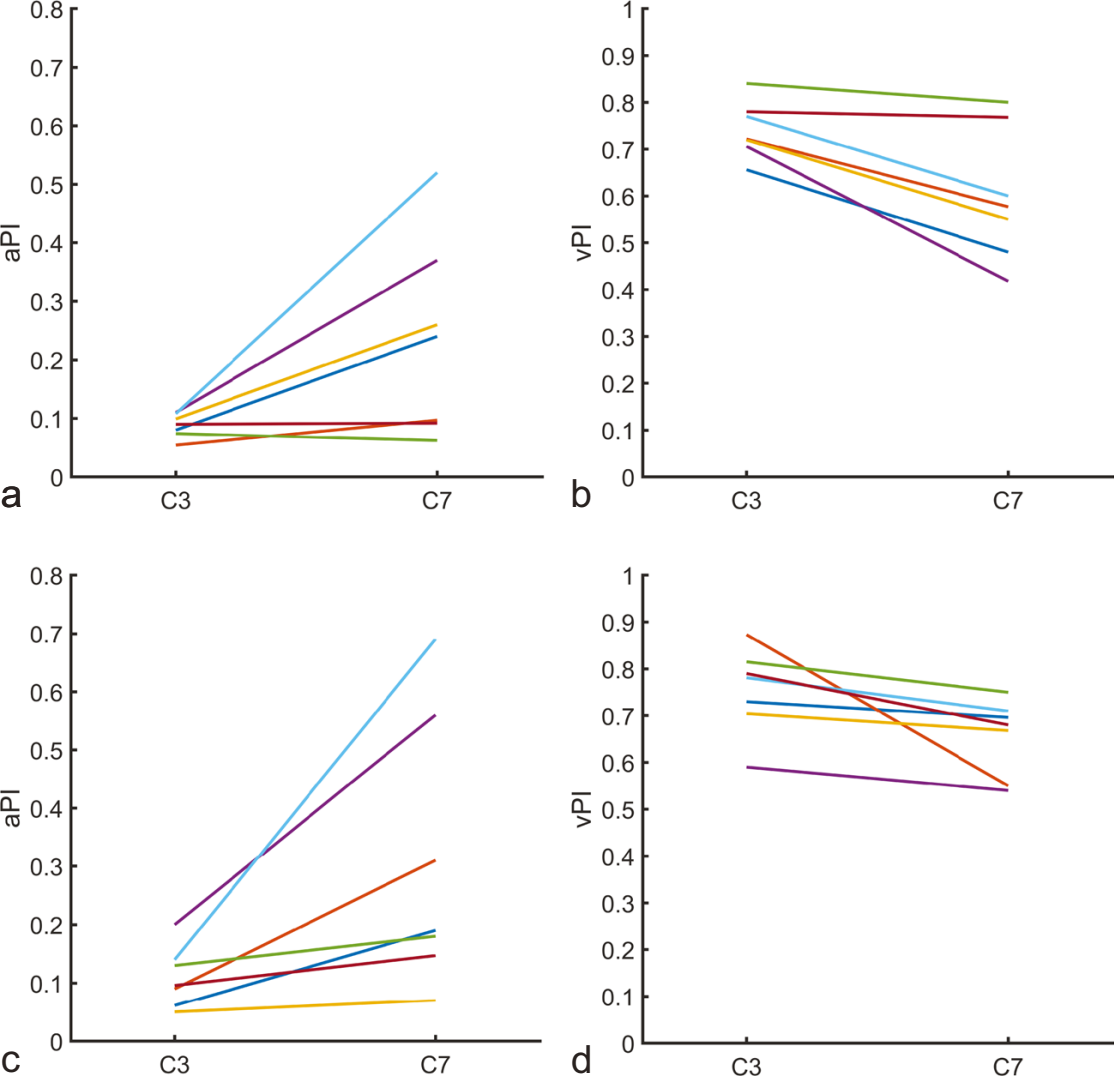
**Comparison between the C3 and C7 segments.** In **a** and **b**, the 2D measurements are given, and **c** and **d**, show the 4D measurements. The colors correspond to the individual participants to facilitate direct comparison between individual measurements, and are similar to those used in Fig. 3. On average, an increase and decrease can be observed between C3 and C7 in lumen area pulsatility index (aPI) and blood flow velocity pulsatility index (vPI), respectively. The decrease in vPI between the C3 and C7 segments as shown in **b** and **d** is comparable to the decrease in pulsatility observed by Schubert et al. and van Tuijl et al.(Schubert et al., 2011; van Tuijl et al., 2020) Similarly, the increase in aPI corresponds well to values reported by van Tuijl et al. Area pulsatility and velocity pulsatility are expected to show opposite trends given the continuity of flow

The blood flow velocity pulsatility and lumen area pulsatility for both the C3 and C7 segment are shown in Fig. 2. The vPI computed from 4D PC-MRI images were comparable to those measured from 2D PC-MRI images. On average, the difference in vPI between 2D and 4D measurements was 0.03 (limits of agreement: −0.17 – 0.23), with 4D measurements on average being 6.8% higher than 2D vPI measurements. Similarly, on average, the difference in aPI between 2D and 4D measurements was 0.05 (limits of agreement: −0.16 – 0.26). The aPI calculated from 4D PC-MRI was on average 45.5% higher than the aPI measured from 2D PC-MRI.

**Fig. 3:**
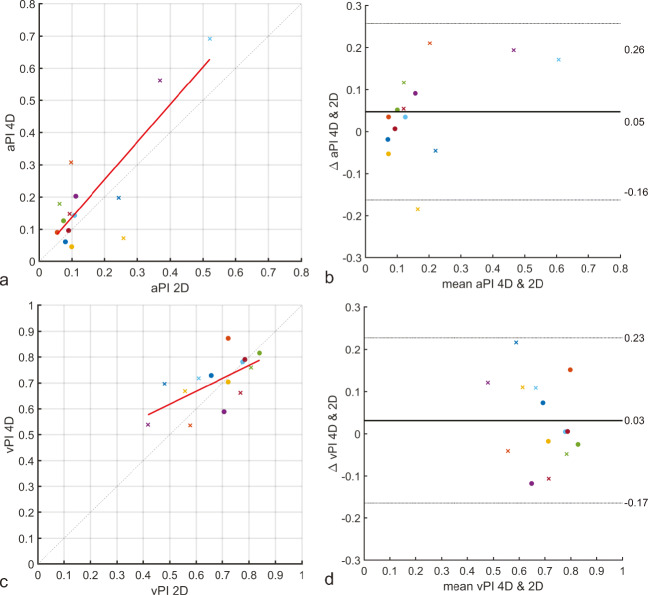
**Comparison between 2D and 4D area lumen pulsatility indices (aPI) and blood flow velocity pulsatility indices (vPI). a** and **c** show the relation between 2D and 4D measurements on the x and y axes, respectively. The y = x line is given by the dashed gray line, and the red line is the line of best fit (***vPI***_**4*D***_ ***=*** **0.50** ***vPI***_**2*D***_ ***+*** **0.37** and ***aPI***_**4*D***_ ***=*** **1.17** ***aPI***_**2*D***_ ***+*** **0.02**). Bland-Altman plots are given in **b** and **d**. Measurements at C3 and C7 level are given by circles and crosses, respectively. The colors of the crosses and circles correspond to individual volunteers and correspond to the color coding used in Fig. 2

Both 2D and 4D measurements, on average, show an increase in lumen area pulsatility between the C3 and C7 segments (Fig. 3). The aPI is 0.08 and 0.21, and 0.10 and 0.24 for the C3 and C7 segments, given the 2D and 4D measurements, respectively. Similarly, a decrease is observed in the vPI between the C3 and C7 segments. On average, the vPI is 0.74 and 0.60, and 0.75 and 0.65 for the 2D and 4D measurements, respectively.

For both 2D and 4D measurements, the blood flow rate was largely preserved between the C3 and C7 segment. In Table 1, the blood flow rate in ml/s is given for all volunteers. On average, the difference in blood flow rate between C3 and C7 was −0.03 ± 0.24 ml/s, and − 0.07 ± 0.21 ml/s given the 2D and 4D measurements, respectively. Even though the blood flow rate was preserved between the C3 and C7 segments, the blood flow rate calculated from 2D was in all cases 20 to 46% higher than the blood flow rate calculated from 4D images.

**Table 1:** Blood flow rate preservation between C3 and C7 segments

ml/s	2D			4D		
	C3	C7	% diff	C3	C7	% diff
1	4.59	4.29	−6.5	3.30	3.05	−7.5
2	4.26	4.48	5.1	3.40	3.27	−3.7
3	5.77	5.56	−3.6	4.02	3.65	−9.1
4	3.99	4.28	7.2	2.92	3.11	6.7
5	4.12	3.85	−6.5	2.95	2.81	−4.8
6	3.22	3.01	−6.6	1.73	1.75	1.3
7	2.94	2.85	−3.2	2.08	2.23	7.6

Rows correspond to individual volunteers. Blood flow rate is averaged over the cardiac cycle. The % difference denotes the difference between C3 and C7, against the blood flow rate at C3

**Fig. 4:**
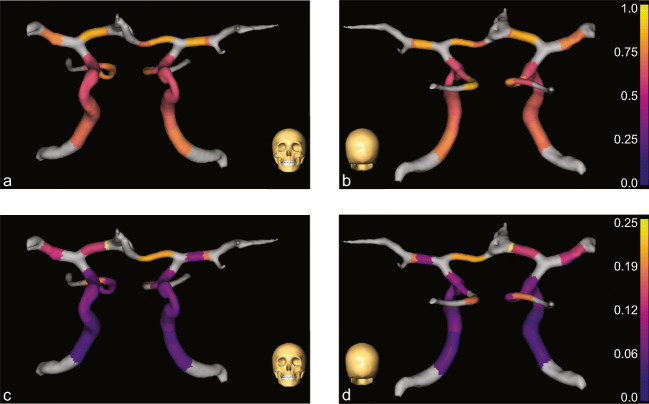
**Blood flow velocity pulsatility (vPI) and lumen area pulsatility indices (aPI) over multiple arteries of the circle of Willis for one volunteer. a** and **c** show a coronal view in dorsal direction, and **b** and **d** show a coronal view in ventral direction. In **a** and **b**, the vPI is given, and in **c** and **d**, the aPI is given for part of the circle of Willis. The bottom most arteries are the internal carotids, that branch of into the posterior communicating arteries/posterior cerebral arteries. The basilar artery is not shown. The internal carotids branch outwards into the middle cerebral arteries, and inwards in the anterior cerebral arteries that join in the middle of the panels. A decrease in vPI can be observed along both internal carotid arteries, whereas the aPI increases. Both the aPI and vPI are high in the anterior cerebral arteries

In Fig. 4 we show vPI and aPI for a large part of the circle of Willis for one volunteer. A decrease in vPI can be observed along both internal carotid arteries, whereas the aPI increases. Both the aPI and vPI are high in the anterior cerebral arteries and in the posterior cerebral arteries. Left and right arteries show similar vPI, whereas there is more variation in aPI.

## Discussion

In this study, we have developed software for measuring the blood flow velocity pulsatility and lumen area pulsatility of the intracranial arteries from 4D PC-MRI images. We have shown that the blood flow velocity pulsatility measurements using our software on 4D PC-MRI images correspond well to measurements on 2D PC-MRI images. A significant decrease and increase in blood flow velocity pulsatility and lumen area pulsatility, respectively, were observed between the C3 and C7 segment of the internal carotid artery. On average, the lumen area pulsatility was 140% higher in the C7 segment compared to the C3 segment, and the blood flow velocity pulsatility was 13.3% lower in the C7 segment compared to the C3 segment. When combining blood flow velocity and luminal area measurements, the blood flow rate was mostly preserved.

The change in blood flow velocity pulsatility between the C3 and C7 segments is comparable to the change in pulsatility observed by Schubert et al.(Schubert et al., 2011). The absolute pulsatility values are however higher in the work by Schubert et al., which can potentially be explained by differences in the definition of the pulsatility index. Schubert et al. used volume flow rates (in ml/min) to calculate the pulsatility index, whereas we used the blood flow velocity. Van Tuijl et al. report similar blood flow velocity pulsatility indices for the C3 and C7 segments (van Tuijl et al., 2020).

Besides the pulsatility and blood flow velocity, the arterial stiffness is an important biomarker for vascular health. There are three commonly used non-invasive ways to derive the arterial stiffness (Oliver & Webb, 2003). The first one derives the arterial stiffness from the time delay in the arrival of the arterial pulse wave. This method has been properly validated, but is commonly used in the larger arteries where the temporal resolution of PC-MRI sequences are sufficient for measuring this delay (Wentland et al., 2014). For the intracranial arteries the temporal resolution of used 4D PC-MRI sequences is too low to measure this. However, Peper et al. have developed a 2D PC-MRI sequence with a temporal resolution that is high enough to measure the arrival time delay between sections of the (internal) carotid artery (Peper et al., 2018). Secondly, the arterial stiffness can be derived from analysis of specific components of the arterial pressure or flow waveform (Oliver & Webb, 2003). Thirdly, the arterial stiffness can be derived by measuring the change in luminal area/diameter given the increase in pulse pressure, i.e. the arterial distensibility. In the current study, we did not possess pulse pressure measurements. However, the observed changes in lumen area pulsatility over the cardiac cycle for the C3 and C7 segments corresponded to values reported by van Tuijl et al.(van Tuijl et al., 2020). The lower lumen area pulsatility at C3 (relative to C7) probably reflects the constrictive effects of the bony carotid canal at that segment of the ICA. The lower lumen area pulsatility is compensated by a higher velocity pulsatility, which explains the opposite trends in vPI and aPI seen in Fig. 2. Van Tuijl et al. also observed a similar relatively large intersubject variation for the aPI on the C7 level when measured from 2D PC-MRI. This intersubject variation is likely a combined effect of both physiological differences and measurement errors. Taking an average pulse pressure of 40 mmHg for healthy volunteers, the aPI at C7 (where the vessel is not hampered by the bony carotid canal, as is the case for C3) corresponds to an arterial distensibility of 0.50%/mmHg, which corresponds well with values reported for the intracranial arteries (Giannattasio et al., 2008; Studinger et al., 2003; van Tuijl et al., 2020; Warnert et al., 2015).

We observed that the measured blood flow rate between the C3 and C7 was largely preserved. This shows that our proposed software correctly captures the variation in arterial lumen area and blood flow velocity pulsatility that exists along the distal end of the carotid artery (Schubert et al., 2011; van Tuijl et al., 2020). In most cases, the blood flow rate was slightly lower at the C7 level. This difference may be caused by the ophthalmic artery, that is connected to the internal carotid at the C6 level. Ambarki et al. show that in healthy young volunteers the average flow rate through the ophthalmic artery is 0.17 ml/s, which might (partially) explain the lowered blood flow rate at the C7 level (Ambarki et al., 2013). The observed difference in flow rate between C3 and C7 are relatively comparable between 2D and 4D, strengthening the idea that this observed difference in flow rate is caused by the ophthalmic artery branch. However, in some cases we observe a (slight) increase in blood flow rate between the C3 and C7 level, which is very implausible in this group of healthy young volunteers (only in patients with severe carotid artery stenosis, this may indicate collateral flow via a reversed flow direction in the ophthalmic artery (Zarrinkoob et al., 2019)). Between 2D and 4D measurements, the flow rate was 20 to 40% lower given the 4D PC-MRI images. This is potentially caused by the difference in spatial resolution between the 2D and 4D PC-MRI acquisitions (in-plane resolution, 2D: 0.19 mm. 4D: 0.78 mm) (Bollache et al., 2016; Stalder et al., 2008). The voxels at the edge of the lumen suffer from more severe partial volume effects in the 4D PC-MRI images. This lowers the image intensity for these voxels, subsequently leading to smaller delineated luminal areas.

The difference in blood flow rate can also be caused by the image intensity threshold used to delineate the arterial lumen. Dunås et al. show that delineating the arterial lumen at an intensity of 20% of the maximum intensity of the complex difference PC-MRI image yields the lowest difference with 2D PC-MRI blood flow rate measurements (Dunås et al., 2019). We delineated the arterial lumen from the background at FWHM intensity value in the PC-MRI magnitude image. However, we used the same isocontours intensity threshold for both 4D and 2D images, whereas Dunås et al. used a commercial tool for the segmentation of the arterial lumen on 2D PC-MRI images. Additionally, the lower temporal resolution of the 4D PC-MRI acquisition compared with the temporal resolution of the 2D PC-MRI acquisition may partially explain the lower observed blood flow rate measured from 4D PC-MRI (Bollache et al., 2016).

(Commercial) software for measuring blood flow rate and pulsatility of the intracranial arteries are readily available for analysis of 4D PC-MRI images (Schrauben et al., 2015; Vali et al., 2019), but commonly assume a static lumen over the cardiac cycle. Software that does allow for dynamic measurement of the arterial lumen is often times tailored to the large extracranial arteries such as the aorta. Our developed software allows for calculation of a dynamic luminal area over the cardiac cycle for the intracranial arteries. In the future, measuring local changes in the arterial stiffness of the intracranial arteries may provide more insight in cerebrovascular disease progression, and may be linked to damage to the brain parenchyma (Birnefeld et al., 2020; Lee et al., 2017; Saji et al., 2016; Zhai et al., 2018).

Even though the acquisition duration is roughly 8 times longer (2 min and 6 s for the 2D PC-MRI image for a single location compared to 15 min and 30 s for all velocity encoded 4D PC-MRI images), the ability to quantify the blood flow over an extended length of the arterial tree is a major advantage of 4D PC-MRI over 2D PC-MRI. Additionally, 2D PC-MRI requires operators to accurately place the imaging field of view, perpendicularly to the vessel, and perform multiple acquisitions to assess (damping in) pulsatility at different locations along the vascular tree. When angulation is incorrect, blood flow measurements are inaccurate. Processing time of the 4D PC-MRI images using our software is also comparable to that of the analysis of 2D PC-MRI images. Besides selecting the vessel of interest, and optionally altering the vessel contours, most image processing is performed automatically by our software.

The presented approach has been implemented with the MeVisLab framework for image processing research and development (version 3.1.1) and various open-source components listed in our repository. The readme-file in the repository includes step-by-step installation instructions to install our tool, which include: installing MeVisLab (from mevislab.de), downloading our source code, and installing the required packages. The tool runs on any recent workstation and supports Windows, Mac, and Linux.

Several limitations have potentially influenced the outcome of our study. First, the relatively low number of included volunteers could potentially influence the average observed effect of changes to blood flow velocity pulsatility and lumen area pulsatility. However, almost all of our volunteers showed a similar direction of change in blood flow velocity pulsatility and lumen area pulsatility between the C3 and C7 segments. Second, in some cases, flow voids were present in the 2D and 4D acquisitions, which challenged the automated contour drawing algorithm. In such cases, manual delineation around the flow void was required. These manual corrections could have influenced our measurements. However, flow voids -if present- were only visible on a few timepoints, for which manual editing was necessary. In most cases, the automated algorithm performed well. Third, the inflow effect of slow flowing blood near the edges of the lumen could have potentially increased the apparent luminal area in the 4D PC-MRI images. We minimized this effect by angulating the 4D PC-MRI acquisition such that the internal carotid below the C3 segment was outside of the field of view. Fourth, the challenging angulation of the 2D PC-MRI on the C7 segment potentially influenced the measurements. However, potential errors in the angulation of our 2D PC-MRI sequence are likely minimal, because blood flow rate measurements at the C7 level were found comparable to those at the C3 level, and the results were comparable to the 4D flow analysis where the perpendicular cross-section was automatically derived from the detected centerline of the vessel.

## Conclusion

Our software allowed for measurement of the blood flow velocity pulsatility and lumen area pulsatility on 4D PC-MRI images. The measurements of our software were validated against measurements performed on 2D PC-MRI images. Given that the flow rate between both evaluated segments of the internal carotid artery were largely preserved shows that this software is capable of measuring the variation in arterial lumen area and blood flow velocity over the cardiac cycle. Our software may potentially aid in identifying changes in arterial stiffness of the intracranial arteries caused by pathological changes to the vessel wall.

## Data Availability

The data that support the findings of this study are available, upon reasonable request, from the corresponding author.

## Code Availability

The developed software that support the findings of this study are publicly available from Github (https://github.com/keesvanhespen/DampingGUI).
